# SiC surface orientation and Si loss rate effects on epitaxial graphene

**DOI:** 10.1186/1556-276X-7-186

**Published:** 2012-03-12

**Authors:** Moonkyung Kim, Jeonghyun Hwang, Virgil B Shields, Sandip Tiwari, Michael G Spencer, Jo-Won Lee

**Affiliations:** 1 School of Electrical and Computer Engineering, Cornell University, 410 Thurston Avenue, Ithaca, NY 14850-2488, USA; 2 Department of Convergence Nanoscience, Hanyang University, 17 Haengdang-dong, Seongdong-gu, Seoul, 133-791, South Korea

## Abstract

We have explored the properties of SiC-based epitaxial graphene grown in a cold wall UHV chamber. The effects of the SiC surface orientation and silicon loss rate were investigated by comparing the characteristics of each formed graphene. Graphene was grown by thermal decomposition on both the silicon (0001) and carbon (000-1) faces of on-axis semi-insulating 6H-SiC with a "face-down" and "face-up" orientations. The thermal gradient, in relation to the silicon flux from the surface, was towards the surface and away from the surface, respectively, in the two configurations. Raman results indicate the disorder characteristics represented by I_D_/I_G _down to < 0.02 in Si-face samples and < 0.05 in C-faces over the 1 cm^2 ^wafer surface grown at 1,450°C. AFM examination shows a better morphology in face-down surfaces. This study suggests that the optimum configuration slows the thermal decomposition and allows the graphene to form near the equilibrium. The Si-face-down orientation (in opposition to the temperature gradient) results in a better combination of low disorder ratio, I_D_/I_G_, and smooth surface morphology. Mobility of Si-face-down orientation has been measured as high as approximately 1,500 cm^2^/Vs at room temperature. Additionally, the field effect transistors have been fabricated on both Si-face-down and C-face-down showing an ambipolar behavior with more favorable electron conduction.

## Introduction

Graphene is a sheet of graphite consisting of sp^2^-bonded carbon atoms [1]. The unique material properties of graphene such as extremely high-carrier mobility, semi-metallic characteristics, and two-dimensional [2-D] very thin sheet of carbon have attracted a great interest and will lead to the development of nanoelectronics [2,3]. The graphene was first obtained by cleaving the graphite, but this drawing/exfoliation method is only useful for the demonstration of scientific or engineering concept rather than a large volume manufacturing [1,4]. The result of exfoliation is not predictable, and the available size is too small (< 100 um) for practical application. In order to obtain a large area graphene, the chemical vapor deposition [CVD] growth on catalytic metals or thermal decomposition of SiC has been extensively studied [5-8]. Large area good quality graphene was produced using the CVD method, but the grown graphene has to be transferred to an insulating substrate since the graphene cannot be used on the metals in most applications [9]. This transfer method needs costly processes and likely causes damages to the grown graphene. Thermal decomposition of SiC produces the so-called epitaxial graphene and has shown high crystal quality [10,11]. Since this epitaxial graphene can be directly formed on an insulating large area substrate compatible with the already established semiconductor processing technique, it is a promising route for commercialization of graphene devices. The epitaxial graphene is grown as a result of Si evaporation at high temperature and can be grown under the ultra high vacuum [UHV] or atmospheric Argon [Ar] environment. The growth under Ar requires higher-annealing temperatures (1,500°C to approximately 2,000°C) for Si to be evaporated overcoming the Ar pressure near the substrate surface [12]. This method reduces the Si evaporation rate and enhances the surface diffusion resulting in the formation of higher quality graphene. However, this technique needs extremely high temperature and may not be compatible with some samples such as SiC epi on Si substrate. The epitaxial graphene can be formed at relatively lower temperature (1,150°C to approximately 1,450°C) in the case of UHV, but the film quality is usually lower than that grown under the Ar at higher temperature (1,500°C to approximately 2,000°C) due to the fast and uncontrollable Si loss. Here, we explore the UHV growth of epitaxial graphene using a face-down configuration. In order to investigate the effects of silicon evaporation rate on the film quality, we designed a growth configuration that would allow the minimization of silicon loss and at the same time provide a way to examine the effects of a much higher rate of Si evaporation. Both the Si-face and C-face of SiC were chemically and mechanically polished [CMP], and the growth was done on both surfaces with the thermal gradient directed towards one surface and away from the other. This arrangement provides a condition for low and high silicon loss at the same time.

## Experiments

**Figure 1:**
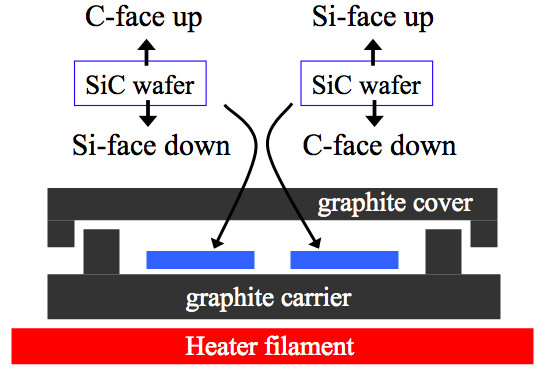
**Diagram of graphene growth configuration**. The diagram showing the growth orientation for graphene on the SiC faces. A graphite cover minimizes the temperature gradient across the wafer during its growth. The thermal gradient is upwards in the diagram.

Growth was done in a conventional-vertical cold wall chamber equipped with a turbomolecular pump. On-axis insulating 6H-SiC was prepared for this study, and both the Si-face and C-face were CMP polished to remove the surface scratches and other defects. The samples were thoroughly cleaned using acetone and methanol before loading and then placed on a graphite carrier. The SiC wafers were contained within a small graphite enclosure on the carrier, and a cap made of graphite covered the enclosure. The samples were placed on the graphite surface within the enclosure such that one face was oriented towards the heater direction and the other oriented away from the heater as shown in Figure 1. Graphene growth was carried out on both faces of the SiC with the Si-face-down (C-face-up) and with the C-face-down (Si-face-up) simultaneously under the same conditions. The chamber pressure varied between lower 5 × 10^-7 ^Torr (idle) and 2 × 10^-5 ^Torr (high-temperature annealing). Sample heating was done using a DC electric power with a graphite filament located at the bottom side of wafer carrier, and temperature was measured by pyrometer. Graphene was grown at 1,450°C for 60 min. The temperature was ramped up to the growth point from 700°C over 1 min and then cooled down with a rate of 15°/min. The grown graphene was characterized by Raman spectroscopy (Renishaw inVia Raman microscope, Renishaw, New Mills, Wotton-under-Edge, Gloucestershire, UK) using 488 nm Ar ion laser, ambient Atomic Force Microscopy [AFM] (Veeco Dimension 3100, Veeco Probes, Ekwill St.

**Figure 2:**
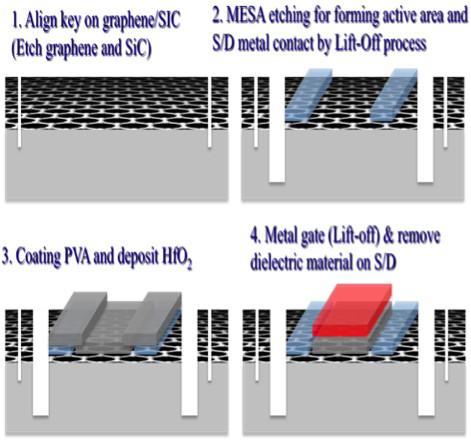
**Simplified schematic of device fabrication on graphene film**.

Santa Barbara, CA). Then, devices were fabricated on these graphene films. Figure 2 shows the simplified schematic of device fabrication with MESA isolation method. First, an active layer for Hall bars and field effect transistor was patterned using an optical lithography and oxygen plasma etching process. This was followed by contact formation using Ti/Au evaporation. Hall measurements with a magnetic field of approximately 0.2 Tesla [T] were conducted in a Van der Pauw configuration using a projected field magnet-probe station and a semiconductor parameter analyzer. Fifty-six nanometer of HfO_2 _was deposited on graphene using the atomic layer deposition at 110°C. Finally, the gate was defined using the optical lithography and e-beam evaporation of Ti/Au (5 nm/150 nm).

## Results and discussions

**Figure 3:**
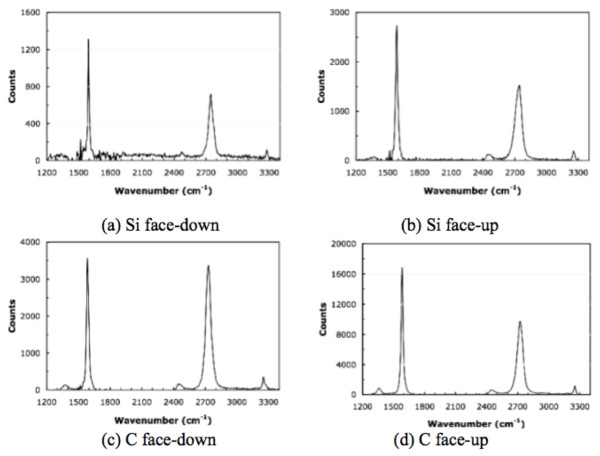
**Raman spectra for the various orientations of the SiC faces**. The Raman spectra for the various orientations of the SiC faces were measured for samples grown at the same time under the same temperature conditions. The underlying spectra for the SiC substrate measured at the same time have been subtracted to produce the graphene results. These samples were grown at the same time in the same carrier at 1,450°C for 60 min. The Si-face-up (**b**) and C-face-down (**c**) spectra are from the two sides of one wafer piece, while the corresponding Si-face-down (**a**) and and C-face-up (**d**) spectra are from a second wafer piece.

**Figure 4:**
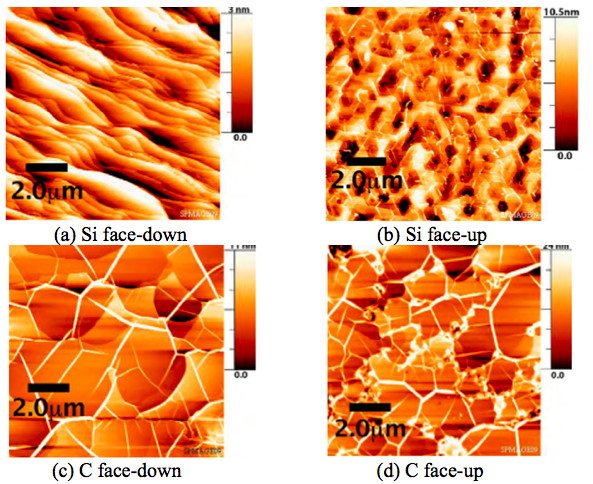
**AFM images**. Images are shown for 10 μm by 10 μm region of the graphene layer surface for various orientations of the SiC faces grown at 1,450°C for 60 min. The height scale is displayed at the right side of each image.

**Figure 5:**
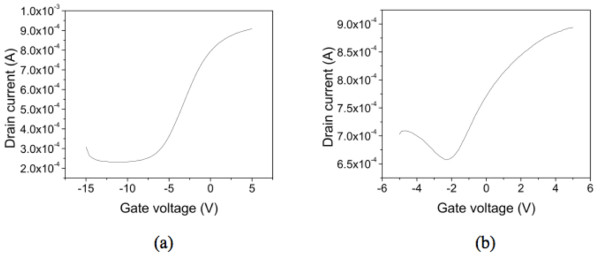
**Transistor transfer characteristics**. In the Si-face-down orientaion graphene (**a**) and C-face-down orientation graphene, gate length/width = 10 um/10 um (**b**).

The signature of graphene as measured by Raman spectroscopy was observed in all the samples. Figure 3a, b, c, d showed the Raman spectra on four surfaces (Si-face-down, Si-face-up, C-face-down, and C-face-up). All the Raman spectra were obtained by subtracting the Raman signal of SiC substrate which was measured before the growth of the graphene. The Si-face-down and C-face-up were from one substrate, and the C-face-down and Si-face-up were from the other. One or two monolayer [ML] to multilayer (up to approximately 30 ML) graphene films were formed on the SiC depending on the face and growth configuration. The thickness of graphene was roughly calculated based on the attenuation of substrate Raman signal [13]. The thinnest film (1-2 ML) was achieved with the Si-face-down arrangement, and the thickest one (approximately 30 ML) was from the C-face-up surface. Larger Raman intensities were measured for the face-up orientations over the corresponding face-down orientations implying thicker films on face-up orientations. Also, the Raman intensities were higher on the C-face surfaces compared to the Si-face ones. In all the cases, sharp G- and 2D-peaks were observed at around 1,590 cm^-1 ^and 2,710 cm^-1^, respectively. The Si-face-down surface showed the narrowest G-peak with the full width at half maximum of 19 cm^-1^. Defect related D-peaks were also found at around 1,350 cm^-1^, and the relative intensity of D-peak and G-peak in the Raman spectra was used to estimate the quality of graphene and the degree of disorders. This I_D_/I_G _ratio has been known to be inversely proportional to the grain size and provides a good estimation for the quality of graphitic materials [14]. The I_D_/I_G _ratio was below 0.05 in the C-face samples, and the ratio was even smaller (< 0.02) in the Si-face ones. Surface morphology was examined using AFM, and the images from the four surfaces are seen in Figure 4. A significant difference in the surface morphology was observed between the face-down and face-up. Samples with the face-down configuration showed much smoother surface compared to the face-ups. It is also noted that the morphology has been affected by the silicon and carbon faces in the downward and upward direction. On the Si-face-down sample, the steps of SiC substrate were clearly observed through the graphene film. However, any step was not observed on the Si-face-up surface, even though the I_D_/I_G _values were very low. Also, pit-like features were formed on the Si-face-up samples resulting in much rougher surface. Large domain-like features and folds were seen on the C-face surfaces, but the SiC steps were not observed. The domain-like features are flatter region surrounded by high boundary (folds). The slightly higher I_D_/I_G _ratios in C-face samples are probably due to the presence of the boundary-like folds. The C-face-up surface had even smaller domain and higher folds than the C-face-down samples. These rougher surfaces of the face-up samples could be attributed to the rapid Si evaporation before reconstructing the SiC surface. Carrier mobility and density were obtained using Hall-effect measurement. Metal contacts were made following the conventional Van der Pauw geometry. The measurements were conducted at room temperature [RT] with a magnetic field of 0.2 T. RT mobility as high as approximately 1,500 cm^2^/Vs was measured from the graphene on Si-face-down sample with a sheet carrier density of 2 to approximately 3 × 10^13 ^cm^-2^. As reported previously on epitaxial graphene, mobility increased as carrier density decreased [15]. Figure 5 shows the front gate transfer characteristics with having 10 um/10 um of gate length/width on Si-face-down and C-face-down graphene layers. A constant source-drain voltage of 0.5 V has been applied, and gate voltage has been swept for the transfer characteristics. An ambipolar behavior has been observed in both cases as observed by Lemme et al. [16]. Specially, transistor on the C-face-down graphene shows more favorable electron conduction than that on the Si-face-down graphene. The threshold voltage was negatively shifted in both devices of the Si-face-down and C-face-down graphene. This can be attributed to the effects of positive fixed charges near the interface (caused by the 'zeroth layer' which occurs during the epitaxial growth of graphene [17]) or fixed charges within insulator layer. Field effect mobility in Si-face-down was acquired from this characteristic, 740 cm^2^/Vs. The degradation of the field effect mobility is likely caused by the dielectric layer on top of the graphene as well as the effects of device fabrication.

## Summary

By comparing the face-down (low Si evaporation rate) and face-up (high Si evaporation rate) mounting method, the optimum growth condition producing a higher quality graphene in a cold wall UHV chamber was found. The use of graphite enclosure and the face-down scheme provide a condition that can reduce Si loss rate and allow higher temperature growth. The best quality film was obtained with the Si-face-down configuration with the I_D_/I_G _ratio below 0.02 with smooth and uniform surface morphology. Electrical properties were characterized by test structure-fabricated and Hall-effect measurements. In transistor characteristics, it shows an ambipolar behavior, and the electron conduction is favored over the hole conduction, which is determined by the polarity of initial majority carriers in graphene layer during the formation of graphene. The Hall mobility at RT is as high as 1,500 cm^2^/Vs, and the value of field effect mobility is close to that of silicon devices. This approach to form a graphene film could be used to grow high quality epitaxial graphene in case a relatively lower-annealing temperature is required.

## Competing interests

The authors declare that they have no competing interests.

## Authors' contributions

MK and JH contributed equally to this work. They carried out the graphene growth and device fabrication/characterizations. VS participated in the growth of graphene and material characterizations. ST participated in the design of devices and its coordination. MS and J-WL conceived of the study and design most of this project. All authors read and approved the final manuscript.
